# Postbiotics: a perspective on their quantification

**DOI:** 10.3389/fnut.2025.1582733

**Published:** 2025-06-04

**Authors:** Gabriel Vinderola, Andrzej Benkowski, Marion Bernardeau, Empar Chenoll, María Carmen Collado, Ultan Cronin, Erik Eckhardt, Justin B. Green, Ignacio R. Ipharraguerre, Rober Kemperman, Christophe Lacroix, Junichi Minami, Martin Wilkinson, Mary Ellen Sanders, Seppo Salminen

**Affiliations:** ^1^Instituto de Lactología Industrial (CONICET-UNL), Facultad de Ingeniería Química, Universidad Nacional del Litoral, Santa Fe, Argentina; ^2^Eurofins Microbiology Laboratories, Madison, WI, United States; ^3^Eurofins Scientific SE, Val Fleuri, Luxembourg; ^4^Normandie Université, UNICAEN, UNIROUEN, ABTE UR4651, Caen and Lallemand SAS, 19, Rue des Briquetiers, Blagnac, France; ^5^ADM Research and Development Center-Valencia, ADM Health & Wellness, Parc Científic U, València, Spain; ^6^Institute of Agrochemistry and Food Technology, National Research Council (IATA-CSIC), Paterna, Valencia, Spain; ^7^Becton Dickinson, Research Centre Ireland, National Technology Park, University of Limerick, Limerick, Ireland; ^8^Department of Biological Sciences, University of Limerick, Limerick, Ireland; ^9^DSM-firmenich Houdan SAS, Houdan, France; ^10^Cargill, Inc., Wayzata, MN, United States; ^11^Institute of Human Nutrition and Food Science, Division of Food Science, Faculty of Agricultural and Nutritional Sciences, University of Kiel, Kiel, Germany; ^12^Lesaffre Institute of Science and Technology, Marquette-Lez-Lille, France; ^13^Laboratory of Food Biotechnology, Department of Health Sciences and Technology, ETH Zürich, Zürich, Switzerland; ^14^International Division, International BtoB Department, Morinaga Milk Industry Co., Ltd., Tokyo, Japan; ^15^Mary Ellen Sanders LLC., Centennial, CO, United States; ^16^Center for Nutrition and Food Research, Faculty of Medicine, University of Turku, Turku, Finland

**Keywords:** postbiotics, quantification, flow cytometry, International Scientific Association for Probiotics and Prebiotics, metabolites, qPCR

## Abstract

A “postbiotic” is *a preparation of inanimate microorganisms and/or their components that confers a health benefit on the host*. To encourage collaborative problem-solving to address the issues related to the characterization and quantification of postbiotics, a working group of academic and industry scientists involved in research or commercial production of postbiotics convened at the International Scientific Association for Probiotics and Prebiotics (ISAPP) 2024 meeting. This paper reports the outcomes of that discussion. Postbiotics are potentially compositionally complex mixtures, leading us to anticipate that full characterization and quantification of all components of a postbiotic product is not feasible. However, confirmation of the identity and quantity of the progenitor microorganism(s), quantification of some of its functional components, and a suitable description of the process of inactivation will be needed to assure the product can be sufficiently described and consistently reproduced. Measurement and quantification must be fit for purpose. Some useful methods include flow cytometry (FC), including innovations such as imaging FC, which has evolved into a mainstream technique suited to quantify inanimate cells, and quantitative polymerase chain reaction, which complements FC by enabling quantification and identity of microbes to the strain level. Other methods can be utilized depending on the complexity, type of microorganisms used (bacteria, yeasts, filamentous fungi), number of strains and cell integrity (intact vs. fragmented). Hence, no ‘gold standard’ methodology - analogous to colony-forming units for probiotics - is envisioned for postbiotics. This perspective focuses on the required microbial composition of postbiotics, not on the optional metabolite components, which can be measured using well-established methods. We propose a decision tree to aid deliberation among different quantification methods for postbiotics under development and being commercialized. We recognize that the evolution of technologies will likely result in future refinement of this decision tree, and we emphasize that our intent is not to prescribe a rigid framework, but rather to provide guiding principles on approaches to quantifying postbiotics.

## 1 Introduction

Postbiotics, which are related to probiotics, prebiotics and synbiotics under the umbrella term ‘biotics’, have emerged as substances that can contribute to host health. The definition of a “postbiotic” is *a preparation of inanimate microorganisms and/or their components that confers a health benefit on the host* (1). The rationale, scope, wording, composition and commercial implementation of this definition were subsequently elucidated (2). Application of this definition to product development and production realities requires addressing some technological challenges. Foremost of these are the clear description of the postbiotic product composition, quantification of the key active components of the final product, and robust standardization of the production process to ensure consistent finished products. Postbiotic progenitor strains must be properly characterized using whole genomic sequencing to confirm proper taxonomy and strain identification. Identifying appropriate methods to sufficiently describe and quantify the postbiotic product is paramount.

At the 2024 meeting of the International Scientific Association for Probiotics and Prebiotics (ISAPP), a group of 32 academic and industry scientists involved in research or commercial production of postbiotics met to address these challenges. Discussion points deliberated during the meeting are reflected in the subheadings below. This paper summarizes multi-stakeholder perspectives, including a decision tree, to provide guidance to postbiotic developers, manufacturers and regulators regarding tools for characterization and quantification of finished products. As a nascent field, we anticipate rapid development of methods for postbiotic quantification and thus our proposals herein are not intended to prescribe a rigid framework but rather to present guiding principles on how to describe and quantify postbiotics.

## 2 Coping with the complexity of a postbiotic

The definition of postbiotics anticipates that the methodology of manufacture and the microbial inactivation process (heat, high pressure, radiation, lysis, or other) resulting in a specific preparation is inherent to the functionality of the product. The final postbiotic preparation must include inanimate microbes, either as intact dead cells, fragmented cells or as cell lysates. Microbial metabolites can be present in the preparation, or not, as is the case when the biomass is extensively washed. Therefore, a postbiotic product can be a complex mixture of functional components.

Such complexity is not unique to postbiotics. Parallels can be drawn to probiotics. A probiotic finished product may be much more complex than what is stipulated by the definition. Although the expected active ingredient of a probiotic product is live microbes, a probiotic finished product will always contain dead cells, which can result from product processing, such as freeze-drying, or natural death during product storage. It may also include fermentation metabolites, if biomass is not extensively washed before freeze-drying (3), or if, for example, the probiotic product is a fermented food. The fraction of dead cells in commercial probiotic products varies, with one conservative estimate of lactobacilli and/or bifidobacteria capsules between 10 and 30% (4). Thus, both probiotic and postbiotic products might be complex mixtures of active ingredients. For probiotics, the live microbial component is regarded as the essential active ingredient characterizing the finished product, typically quantified by colony forming units (CFU), even if other active ingredients (metabolites, dead cells, cell fragments) may also contribute to the overall health benefit.

Given the diversity of all ingredients potentially present in a postbiotic preparation, it may be difficult to pinpoint one or a small set that comprises the active ingredient(s). Then, stipulating the key reference component(s; metabolite, cell-wall beta-glucan, or other) may be a pragmatic decision, rather than one solely based on mechanistic insights. When possible, quantifying based on inanimate cells is a judicious option. If not, one or more molecular factors, either specific (a metabolite, for example) or categorical (such as protein content), could be measured. Such quantitative assessment markers need to be used in conjunction with a complete description of the method of manufacture in order to ensure reproducible postbiotic preparations.

Probiotics have been historically standardized based on CFU, although we recognize limitations of this method, especially its applicability to multi-strain or multi-species products (5). Flow cytometry (FC) is increasingly being used to quantify probiotics (6). The potential complexity and diversity of postbiotics may dictate that one ‘gold standard’ method will not emerge for postbiotics, but instead, a variety of analytical tools adapted to encompass the complexity of the finished product will be needed. For example, in the case of a postbiotic product that delivers a single inanimate microbe devoid of metabolites, the approach will be different than for a product that delivers multiple microbes, or fragmented cells, with metabolites. The development of future guidelines and standards for the characterization and quantification of postbiotics must take these issues into account.

## 3 Measurement and quantification must be fit for purpose

This section explores the importance of tailoring approaches to the measurement and quantification of postbiotics for purposes defined by the target end-user. The types and numbers of measurements made should match the purpose they serve. To avoid excessive or inappropriate measurements, we discuss measuring (assigning a number) and quantifying (expressing a quality based on numbers) active substances in postbiotics. The utility of these measurements depends on the end-user, such as designers, manufacturers, regulatory bodies, healthcare professionals, or consumers. The value of numbers vary in nature, use, and purpose, so it is necessary to discuss the intended use and recipient before establishing methods. Questions about measurement data include: What purpose does the measurement serve? What are the expected results? Can they be interpreted and understood as intended? Who should conduct the quantification? In what capacity and why?

From an industrial perspective, it is necessary to differentiate numbers from research and development (R&D) and those from manufacturing. During R&D, methods and data result from internal decisions to facilitate formulation. Data inform goal setting, candidate selection, dose/effects alignment, and validation of beneficial effects in clinical studies. These numbers must be linked to what researchers aim to measure and evaluate since they coordinate researchers’ actions (selection, rejection, validation). Scientific techniques and methods are chosen for their precision and accuracy in agreement with markers of interest. Table 1 shows the application of various techniques aiming to reflect tested active postbiotic substances or measure postbiotics’ effects *in vitro* or in preclinical models. The choice of technology rests with the scientist. Considering the diversity of potential active substances and mechanisms supporting beneficial effects, there will be many numbers and associated technologies helpful in characterizing and validating postbiotics at the research level. However, these numbers may not mean much to non-scientists and non-professionals and are insufficient to quantify postbiotic products.

**Table 1:** Different techniques used to characterize functionality of postbiotic products.

Postbiotic described in indicated reference	Progenitor strain	Activity	Postbiotic component	Quantification technique	Component/activity measured
Park et al. (40)	*L. rhamnosus* GG	Inhibition of virus-mediated inflammatory responses in HT-29 cells	Cell lysates (mechanical disruption)	Real-time qRT-PCR	Interleukin (IL)-8
			Chemically-extracted peptidoglycan	ELISA	Polyinosinic:polycytidylic acid-induced phosphorylation of mitogen-activated protein kinases
			Heat killed (80°C/2 h) cells	Western Blot Analysis	Activation of NF-κB
Jeong et al. (41)	*L. plantarum* KM2	Muscle atrophy in mice by regulating gut microbiota	Heat-killed (90°C for 1 h) *Lactiplantibacillus plantarum* KM2 with its supernatant	qRT-PCR	Expression of genes associated with skeletal muscle degradation
				Shotgun sequencing	Relative abundance of gut microbial population
Magryś et al. (42)	*L. plantarum* 299v and *L. rhamnosus* GG	Protein extracts secreted by *Lactobacillus* spp.	Heat-killed (90°C for 2 h) cells	Bradford	Protein concentration
		Immune response	Protein extracts from the supernatant	Cytokine (ELISA)	IL-18, IL-10
				Mortality assay	Cytotoxicity

Once developed, postbiotics must be manufactured in a reproducible manner. An adequate description of the manufacturing process is also important to assure that the preparation made in production matches what was used in efficacy studies. Table 2 describes approaches applied for process control in postbiotic production. Their utility is to measure product conformity and batch-to-batch variation, and they can be used as key performance indicators (KPIs). KPIs evaluate performance, efficiency, and quality, monitoring productivity (yield rate, production rate), quality (scrap rate, non-conformity rate), and deadlines. They inform decision-making and performance assessments, helping to reject, accept, or improve production. KPIs are mainly used for continuous improvement and profitability at production sites, and have little meaning for regulators, healthcare professionals, or consumers.

**Table 2:** Some examples of manufacturing process control indicators used in postbiotic production.

Production step	Purpose	Indicator	Technology
Inoculation	Verify strain purity and identity to confirm that the correct strain is propagated and free of mutations	Strain purity Strain identity	Classical microbiology techniques, such as colony morphology and API identification system. Genetic identification techniques, including whole genomic sequencing of progenitor strain, DNA extraction from the biomass, and PCR compared to the control strain
Fermentation	Verify values of fermentation parameters	Final pH Production and consumption rates Final biomass	pH meter and sensors Expressed in g/l Expressed in mass or CFU/ml
	Batch to batch variation	Protein content Final biomass	Kjeldahl CFU/ml using agar count plate
Inactivation	Verify efficacy of the inactivation process	Microbial cell cultivability	CFU/ml before and after inactivation process using agar count plate
		Microbial cell metabolism activity	Flow cytometry combined with live-cell metabolic activity fluorescent markers of cell metabolism.

Numbers used as KPIs differ from those generated by quality control (QC). QC identifies defects to ensure defective products do not reach the public, guaranteeing product composition and safety. At this stage, technologies and data must meet standards set by authorities. Measures are used to judge, evaluate, find agreement, give authorization, assess effectiveness, and compare situations. In postbiotics, an example of QC quantification could be the absence of *Salmonella* expressed as CFU/g (microbial product release analysis), and net quantity.

At the QC stage, numbers move from the private company sphere to the public domain, involving regulatory oversight and consumers. Quantifying is not neutral; it requires agreement, common rules, and understanding among stakeholders. It relies on social and political conventions established before counting. Agreements concern the product (to be quantified) or the procedure (of quantification). Without agreement, results are contested. For postbiotics, it is crucial to define “inanimate microorganisms,” meaning those that cannot generate energy or grow. A deliberate inactivation step is required (1), but not all methods achieve complete inactivation. Regulators should set limits on live microorganisms remaining after preparation to ensure health benefits come from inanimate cells. Recently, the Australian Therapeutic Goods Administration released guidelines for using *Akkermansia muciniphila* in listed medicines^1^. The ingredient is only to be used in a medicine where Qintet Pharmaceuticals Pty Ltd. is the sponsor or has given written authorization to the sponsor. The maximum daily dose should not exceed 34 billion non-viable cells of pasteurized *Akkermansia muciniphila* and must contain fewer than 10 CFU/g of viable cells. In the European Union, EFSA set the maximum count of live cells to less than 10 CFU/g (7).

The regulator provides guidance on quantification conventions, but these are not universal. While the need to quantify is generally recognized, principles and procedures vary. Preferences for standardized and uniform quantification processes exist, but still different global authorities may have different approaches. The European Food Safety Authority system may differ from the United States Food and Drug Administration or China’s Food Contact Materials. Quantification decisions by regulators will depend on their delegated authority and will typically consider fairness, coordination, creating a common language, and building trust. Quantification focused on the number of inanimate microorganisms in postbiotics may make analytical sense, but consumers may have difficulty grasping the idea that a dead entity is efficacious.

Understanding the unique nature of a particular postbiotic can be important both to protect intellectual property and inform mechanistic underpinning of observed efficacy. For example, two different postbiotics might use *S. cerevisiae* but, if manufactured differently, they exhibit different health benefits. A manufacturer could choose unique quality parameters to differentiate its postbiotic.

At the consumer level, the decision to purchase a postbiotic is likely informed by understanding the source, nature and benefits of the product. A postbiotic product label should clearly identify what the product is and is not, avoiding confusion with probiotics and prebiotics. Determining what statements of quantity to put on a product label must balance simplicity and comprehensiveness. The ability to link the potency and composition stipulated on the label with the research documenting health benefits must be considered. In the absence of clear regulations or industry standards, multiple quantification references could confuse consumers.

## 4 The evolving field of flow cytometry as a quantification tool

FC is a rapid, real-time, high-throughput technique based on the measurement of individual cells’ light scatter and fluorescence emission as they flow past one or more lasers. The power of the technique rests on its ability to take multiple measurements from thousands of individual cells per second. In addition to its speed, FC can be adapted to measure heterogeneity in a sample of cells and to quantify and characterize rare events.

Invented 60 years ago, an acceleration of technological developments in FC in recent years is of direct relevance to the quantification and characterization of postbiotics. These developments include: improvements in hardware (smaller, more efficient lasers, more sensitive optics and detectors, small-particle detection), the expansion of available reagents (new families of fluorescent dyes appearing almost monthly), and the appearance of spectral cytometry (8–10). Another recent development, imaging cytometry, is in the process of shedding its status as a niche application, with five new imaging cytometers being released in 2024. Furthermore, a plethora of easy-to-use bioinformatics tools, some of which include machine learning abilities, capable of dimensionality reduction of complex data and clustering of cell populations, place very powerful analytical tools in the hands of even the most novice user (11, 12).

Fluorescent markers can be applied to cells such that the following can be detected and measured through cytometry: broad molecular species such as DNA, proteins, lipids, cell wall sugar moieties, among others; metabolic processes such as the redox state of the cell membrane or enzyme activity, and cell-type or species-specific markers. It is this latter category of marker that has grown into the workhorse of the cytometry performed in the immunology and cancer research fields, and, overwhelmingly, when referring to the specific tagging of cell- or species-specific markers, FC using antibody-coupled probes is what comes to mind (13).

While immunologists have an arsenal of thousands of commercial marker-specific antibodies at their disposal, the availability of off-the-shelf species-specific antibodies continues to present challenges to the scientists interested in detecting microbial species beyond much-studied ones such as *Escherichia coli, Listeria monocytogenes*, and *Staphylococcus aureus*. The state of the art in the immunology field sees instances of a large range of commercial markers being applied to individual samples. For example, Konecny et al. (14) applied 50 commercial markers to their cells and harnessed the power of spectral FC to perform a deep phenotyping of the human immune system. However, such a plethora of commercial antibodies is not available to those interested in the enumeration of typical postbiotic organisms. An alternative to fluorescent antibodies is fluorescence in-situ hybridization (FISH), which involves the fluorescent tagging of DNA probes targeted to sequences of chromosomal DNA, rRNA or mRNA down to the strain level (15). Antibody tagging is generally thought to provide resolution down to the species level. A further category of highly specific markers that can be utilized in FC are aptamers. These nucleic acid-based molecular recognition elements demonstrate similar specificities and affinities as antibodies but demonstrate a number of advantages over antibodies, including increased thermal stability, reversible target binding and a process to generate that does not require the sacrifice of experimental animals (16).

Quantification of postbiotics can broadly be grouped into single or multiple strain categories. For single strains, the degree of complexity of sample preparation and staining required depends greatly on the matrix (a supplement or a food). For a postbiotic having only a single strain, a bright fluorescent dye which binds to a “generic” cell component such as DNA, proteins, or cell wall components will suffice to provide a high enough fluorescent signal-to-noise ratio in order to elevate the stain’s fluorescence from the background. The sensitivity of FC detection is a function of instrument noise and sample matrix interference (see ISO 19344 as a jumping-off point for such an approach). The dye SYBR Gold™ (Thermo Fisher, Waltham, Massachusetts, United States) is useful for such purposes (17).

For detecting a specific strain in a background of other strains, a staining method that can distinguish between the strain of interest and background strains must be employed. While strain-specific antibody or FISH probes are suitable, a lectin, a combination of lectins, or a combination of lectins and DNA and protein makers may also be useful, as shown by Holm and Jespersen (18).

For the quantification of multiple strains in a multi-strain mixture, applying a cocktail of species-specific antibodies, FISH probes or a mixture of the two may be needed. Since the antibodies or FISH probes required might not be commercially available, third parties might be needed to design, create and validate a panel of markers suitable for the application. This can be a lengthy and expensive process.

Two alternatives involve recent advances in FC – the ability to measure and characterize a cell’s autofluorescence (native fluorescence) in the context of spectral cytometry and imaging cytometry. Spectral FC has been shown to be capable of discriminating between strains of the same species which were treated with different levels of gentamicin (19). Since much of a cell’s autofluorescence is derived from metabolically important molecules such as tryptophan, FAD and other flavins, and NADH, the measurement of a cell’s autofluorescence using spectral cytometers holds promise for the designation of a cell’s viability status (20). A good candidate for using autofluorescence is the cyanobacterium *Arthrospira platensis* (Spirulina), a species which shows promise as a supplement in a variety of areas, including the reduction of metabolic syndrome (21). *A. platensis* is remarkably autofluorescent—to the extent that early researchers posited the species as a source of the fluorochrome, allophycocyanin, which is widely used in FC (22).

In imaging cytometry, not only are images recorded for every detected cell, but through the instrument’s image analysis capabilities. A multitude of derived parameters can be measured per cell, putting into numerical format characteristics that can be used to differentiate strains, physiological states or cell integrity (10). Image cytometry may be useful for strain identification, even in challenging samples containing cellular debris and non-target organisms. Masking and multichannel fluorescence imaging can be applied to classify filamentous microbes on the basis of the number of nuclei detected as well as the measurement of the metabolic activity (23).

For the flow cytometric analysis of any microbe from a food sample, the most challenging aspect of the workflow is sample preparation (6). Very often, the food matrix contains interfering particles of similar size (in terms of light scatter and intrinsic fluorescence) and number of the bacteria or yeast being measured, difficulting their quantification. While there is no universal method for preparing clean, single-cell microbial suspensions from food samples for FC analysis, guiding principles include the removal of as much interfering particulate matter as possible (through filtration, centrifugation of chemical or enzymatic treatments), the staining of the strain of interest with as spectrally unique and bright a dye as possible, and the optimization of cytometer settings (multiple thresholds, thresholding on fluorescent parameters). With recent innovations in FC instrumentation and software, it is also possible to use autofluorescence subtraction to minimize interference from food matrix particles (24), image cytometry to remove debris/lipid droplets/starch grains from the analysis, and bioinformatic tools to “recognize” and disregard food debris particles. Table 3 illustrates some examples where different FC techniques were used to quantify postbiotics.

**Table 3:** Examples of quantification of postbiotics using flow cytometry.

Microorganism(s)	Product tested	Staining regime	Cytometer used	Comments	Reference
*Bacillus subtilis* CNCM I-2745, *Bacillus licheniformis* NRRL B-67649*, Bacillus pumilus* NRRL B-67648 and *BBacillus velezensis* NRRL B-67647R	Cell cultures	LDS751 and SYTO24	Attune® NxT Acoustic Focusing Cytometer (Thermofisher)	Method not specifically applied to enumeration of postbiotics, but does show promise of being able to detect non-viable spores and trace vegetative cells. Method was confirmed by cell sorting.	(43)
*Lacticaseibacillus paracasei*	Heat-killed *Lacticaseibacillus paracasei* lyophilized postbiotic	SYTO24 and PI as per ISO 19344 Protocol B	Attune® NxT Acoustic Focusing Cytometer (Thermofisher)	Demonstrated that ISO 19344 can be adapted for postbiotics; data compared well with microscopy counts; could distinguish bacteria in product from maltodextrins.	(44)
*Lactiplantibacillus plantarum, Lactocaseibacillus rhamnosus Lacticaseibacillus casei*, *Bifidobacterium breve*, *Bifidobacterium longum, Bifidobacterium animalis* subsp. *lactis.*	Cell cultures	TO and PI from the BD Cell Viability Kit with liquid counting beads (BD Biosciences, Cat. no. 349483)	FACS Calibur (BD Biosciences); Cytoflex (Beckman Coulter); Attune® NxT Acoustic Focusing Cytometer (Thermofisher)	Robust ring test method carried out in three companies; shows that the BD kit commonly used for probiotics can be applied to postbiotics.	(45)
*Lactobacillus* spp., *Enterococcus* spp., Bacteroidaceae/Prevotellaceae, *Clostridium histolyticum*, *Bifidobacterium* spp., among many others	The review focussed on gut microbiota, but methods could be applied to a variety of samples.	FISH probes (exhaustive list of strain-specific probes given).	N/A	A review which makes the case for FISH-FCM technique being capable of detecting and quantifying many strains of bacteria.	(46)
*Lacticaseibacillus rhamnosus* CRL1505	Cell cultures	TO and PI from the BD Cell Viability Kit with liquid counting beads (BD Biosciences, Cat. no. 349483)	FACS Calibur (BD Biosciences)	In this study, flow cytometric determinations revealed the great impact that growth conditions have on the cellular integrity of Lr-CRL1505 and how specific production conditions lead to a product containing high PolyP.	(47)
*Lacticaseibacillus rhamnosus* ATCC 53103	Cell cultures	CFDA and PI	CoulterEPICS XL-MCL (BeckmanCoulter)	The method could be used to assess degree of cellular intactness of heat or pressure-inactivated cells.	(48)
Review: multiple strains	Multiple products	Multiple stains listed and referenced	N/A	This review is a good source of information on stains that can be used for enumeration of postbiotic strains.	(49)

## 5 Quantitative and digital polymerase chain reaction assays as quantification tools

Quantitative (or real-time) polymerase chain reaction (qPCR) and digital PCR (dPCR) assays use DNA (or RNA) and specific primers and fluorescent probes to simultaneously amplify and quantify the target molecule in real-time. The techniques can also be utilized to quantify postbiotic preparations with accuracy and precision and are increasingly recognized as highly favorable methodologies for this purpose (25). The same approaches for probiotic method development and validation using qPCR/dPCR (26–29) can be applied to the quantification of inanimate microorganisms. Notably, one of the distinct challenges in quantifying probiotics or living cells by qPCR/dPCR is the requirement for optimized viability pretreatment using photoreactive dyes such as propidium monoazide (PMA) or ethidium monoazide (EMA) to inhibit the amplification of dead and damaged cellular DNA based on cell membrane integrity (29).

The capacity to design assays with species and strain specificity is a significant advantage of these PCR-based techniques. It has been demonstrated that probiotic strains can be selectively enumerated in a blended material (in the presence of other probiotics) using qPCR (30, 31), which extends to postbiotic cells incorporated into similar blends with other postbiotic strains. Further downstream, during the production process, qPCR can be used to selectively enumerate postbiotic strains with additional matrix complexities (32), although certain substances and ingredients are known to cause inhibition (33). The ability to generate data in real-time also allows for the evaluation of production processes, such as microbial growth kinetics (34) and inactivation optimization. Multiplexing qPCR assays can also yield selective, rapid, and accurate enumeration of multiple targets in a blend (35).

Challenges associated with testing postbiotics by qPCR/dPCR include the upstream necessity for careful design and validation of primers and hydrolysis probes to ensure exclusive amplification of target DNA (36). This becomes particularly crucial once the material is lyophilized and blended with excipients. A postbiotic quantification assay using qPCR also requires the generation of a standard curve to estimate the number of cells present. The application of this standard curve may be limited to certain types of samples, limiting the scope of the test. Ensuring proper reaction efficiency and performing thorough method validation at the matrix category level can help overcome these challenges. Digital PCR allows for the absolute quantification of DNA or RNA molecules by separating a sample into many small partitions either by droplet formation or on a micro-well chip and does not require using a standard curve to produce a quantitative result representing the concentration of DNA or RNA copies present. The enumeration methods for postbiotics using dPCR require the same level of validation at the matrix category level as those employed in qPCR.

Early in 2025, the China Nutrition and Health Food Association released a “tuan biao” or Industry Standard for quantifying postbiotics that includes a specific qPCR method to detect inactivated *Bifidobacterium lactis* subsp. *lactis*^2^. While this development draws attention to the utilization of qPCR for postbiotic quantification, it does not provide procedures for additional strains or targets and thus has limited value for broader industry adoption.

The composition and industrial processes for the inactivation of the postbiotic should be considered when determining the appropriateness of qPCR/dPCR as a quantification tool. The final number will be a cell count based on the number of copies of DNA; therefore, alternative markers described elsewhere to establish quality parameters of postbiotic preparations, such as the short chain fatty acids production, need not be applied. The process of inactivation has the potential to degrade or fragment the cellular DNA, and fragmentation of DNA can affect the accuracy of the quantitation by PCR (37). Since free DNA in the preparation can cause inaccuracies in cell counts (38), using PCR would require that inactivated cells maintain a degree of structural integrity. Intact, inactivated cells are ideal for quantification by qPCR/dPCR, but in certain industrial applications, it is feasible to enumerate cell fragments as a quality control benchmark as long as the method is validated and fit-for-purpose. The number of species in a blend of postbiotics and the ability to distinguish and quantify each individually is limited by the specificity of the primer/probe design. These factors warrant careful consideration during the development of novel postbiotics and assessment of their quality and efficacy. Table 4 illustrates some examples where different qPCR techniques were used to quantify postbiotics.

**Table 4:** Examples of quantification of postbiotics using qPCR.

Microorganism(s)	Product tested/scope	qPCR instrument used	Reference
*Streptococcus oralis* CECT 907 T *Streptococcus gordonii* ATCC 49818 *Veillonella parvula* ATCC 10790 *Fusobacterium nucleatum* DSM 20482 *Prevotella intermedia* NCTC 13070	Oral biofilm	LightCycler® 480 II (Roche Diagnostics, Penzberg, Germany)	(50)
*B. lactis* ssp. *lactis*	Postbiotic suspension and food with added postbiotics	Not specified	(59)
*Lactobacillus acidophilus* group	Milk	7,500 fast real-time PCR system (Applied Biosystems, Foster City, CA, USA)	(35)
*B. animalis* ssp. *lactis* *Lactobacillus acidophilus* LA-5	Lyophilized product	MX3000P (Stratagene, La Jolla, CA, USA)	(51)

## 6 Choosing a quantification technique for postbiotic commercial products

**Figure 1:**
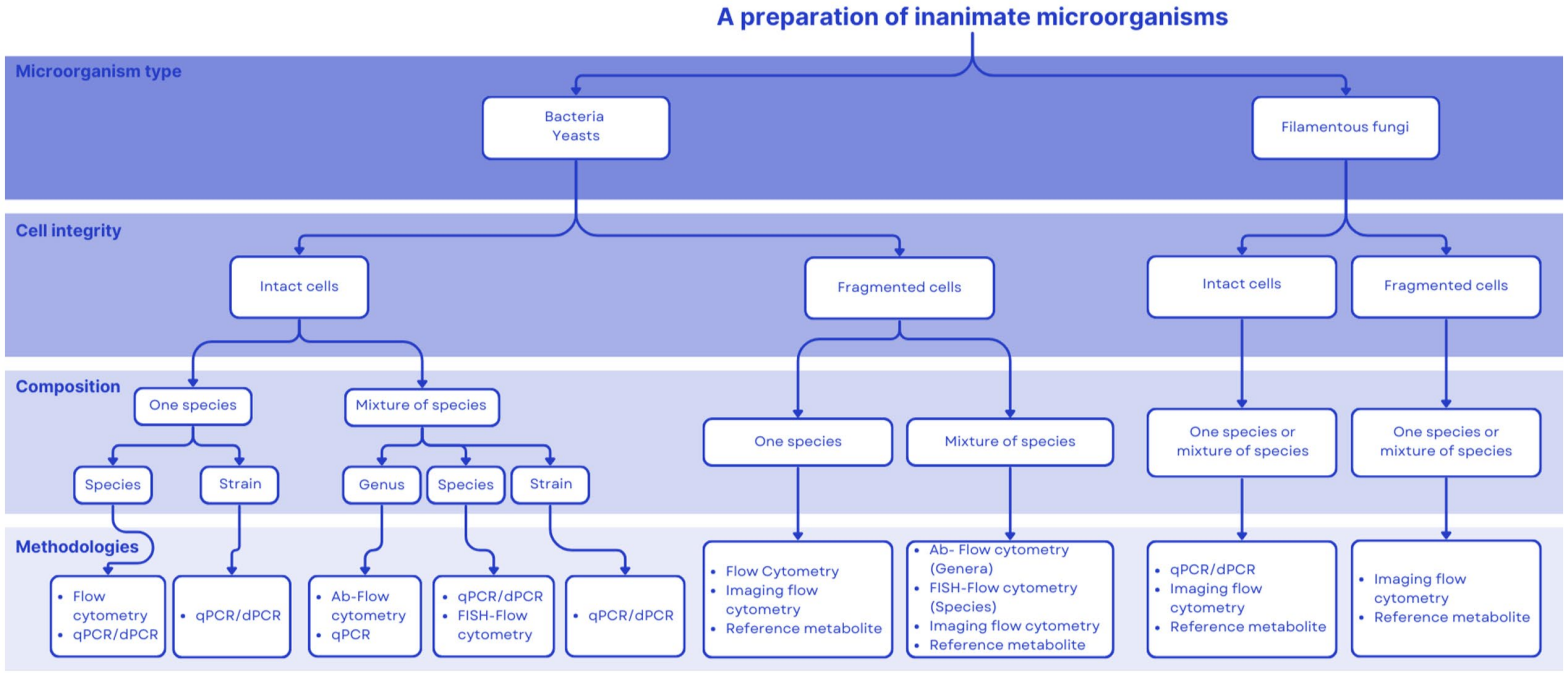
Decision tree to help decide among different quantification approaches for postbiotics.

Figure 1 proposes a decision tree aimed at assisting in the choice of an adequate quantification technique for commercial products. Aspects considered include the number, type and final cell integrity of microorganisms used to formulate the product. This tool will hopefully be useful during the product development pathway. Our intention is for this tool to be useful during the product development pathway. This paper is meant to propose quantification paths for postbiotics as defined by ISAPP (1), which has been elucidated previously (2). This definition focuses on inanimate microorganisms, either inactivated intact whole cells or cell fragments. The purpose of Figure 1 is to present current technologies available to quantify microbial cells. A postbiotic preparation may also contain microbial metabolites, but their presence in the final product is not mandatory. Therefore, a comprehensive characterization of a postbiotic product should comprise, at a minimum, the quantification of inanimate cells or cell fragments (as addressed in this paper), and if relevant, the characterization (identification and quantification) of metabolites produced by the progenitor strain/s before inactivation. The latter may be accomplished by HPLC or mass-spectroscopy technologies, as described elsewhere. Table 5 provides examples of commercial postbiotic products and depicts the approaches used to characterize and quantify them. All the listed products deliver inanimate microorganisms - bacteria, yeasts or fungi - composed of single or multiple strains, either as intact or fragmented cells.

**Table 5:** Characterization and approaches to quantification of some postbiotic products present in the market.

Brand name	Microbe	Progenitor strain(s)	Inactivation method	Cell integrity	Biomass quantification	Does the product contain metabolites?	Metabolite(s) quantified?	Other characterization	References
Safmannan ®	Yeast	*Saccharomyces cerevisiae* (var. baker’s yeast)	Lysis and separation	Cell wall fragments	Dry matter determination	Yes, residual	Yes, mannan and B-glucans polysaccharides, total protein	Microbial contamination	(52)
ES1 HT	Bacteria	*Bifidobacterium longum*	Heat treatment	Whole cells	Counts before HT and flow cytometry	No	No	Number of viable cells and microbial contamination	(53)
BPL1® HT	Bacteria	*B. animalis* subsp. *lactis*	Heat treatment	Whole cells	Counts before HT and flow cytometry	No	No	Number of viable cells contamination	(54)
Humiome ® Post LB	Bacteria	*Limosilactobacillus fermentum* and *Lactobacillus delbrueckii*	Heat treatment	Whole cells	Flow Cytometry	Yes	No	Number of viable cells and microbial contamination	(55)
EpiCor®	Yeast	*S. cerevisiae* (var. baker’s yeast)	Heat treatment	Whole cells with some fragments	Not determined, although protein content is measured which correlates to yeast content	Yes	Yes, total polyphenol, total fiber and and total protein contents	Fourier transform near infrared spectroscopy	(56)
AO.biotics	Filamentous Fungi	*Aspergillus oryzae*	Pulse-combustion drying	Mycelia debris	Milligrams before drying	Yes	Yes, enzymatic activity, mannan and galactose containing oligosaccharides	Absence of viable cells and microbial contamination	(57)
LAC-Shield™	Bacteria	*L. paracasei* MCC1849	Heat treatment	Whole cells	Direct cell count	No	Not applicable	Immune-modulation	(58)

Additionally, once quantification methods are determined, it might be worthwhile for product developers to consider incorporating third-party verifications in their development process. Such approaches are valuable tools for improving consumer confidence in the quality of products once on the market. This concept was discussed in relation to probiotic products (39), but is especially valuable to products that can enter the marketplace without premarket approval from regulatory authorities. In the United States, for example, this would include all dietary supplement products. In short, there are different third-party entities [see (39) for a list] that audit the production process for compliance with good manufacturing standards and assure final products conform to product labeling specifications.

## 7 Regulatory frameworks for postbiotics

The concept of postbiotics is absent from many regulatory frameworks globally. Below, examples of regions that have developed official communications on postbiotics are given.

Health Canada made an early recognition of the term ‘postbiotics’ in a presentation at a scientific meeting held in Chicago in 2023 (personal communication). In Canada, postbiotics fall under the Natural and Non-Prescription Health Products Directorate. At present, there is only one entry for the word postbiotics in the Health Canada webpage,^3^ where it is stated that “gut modifiers as livestock feed are products that, once fed, have a mode of action in the gastrointestinal tract of an animal. The gut modifier category can encompass a variety of feed ingredients; these ingredient types may include, but are not limited to viable microbial strains, prebiotics, postbiotics, enzymes, organic acids and essential oils.” However, no further indications of the meaning of the term postbiotic, nor their use in products for human use, are stated on the website.

As commented above, in January 2025 the National Institutes for Food and Drug Control in China released an industry standard for quantifying postbiotics, using this term to refer to inactivated microbial cells. The standard suggests the use of FC to measure postbiotics composed of inactivated cells of lactic acid bacteria. In addition, a fluorescent quantitative PCR detection method was included for inanimate *Bifidobacterium lactis* cultures.

The TGA (Therapeutic Goods Administration) is the Australian body that regulates medicines, medical devices and biologicals. The TGA recently published a guidance to provide information for applications relating to microorganisms as active ingredients for use as new substances in the listed medicines (the category which includes the majority of dietary supplements marketed in Australia), or as active ingredients in registered complementary medicines (RCM).^4^ Listed medicines and RCM containing microorganisms as active ingredients are generally referred to as probiotics or postbiotics. For the purpose of this TGA guidance, microorganisms are whole and intact cells of bacteria and fungi (including yeasts) that are live or non-viable. This guidance is intended for the premarket assessment of new live and whole/intact non-viable microorganisms potentially used as probiotics and postbiotics. Interestingly, the guidance does not include cell fragments, which have different pharmacokinetics within the gut. It is worth noting that Australia is part of the ACCESS Consortium, consisting of Australia’s TGA, Health Canada, the UK’s Medicines and Healthcare products Regulatory Agency, Swissmedic from Switzerland and Singapore’s Health Sciences Authority. However, it is not yet known whether the ACCESS Consortium will take inspiration from the Australian guidance.

Products that deliver non-viable microbes with health purposes are available and regulated around the world, yet the term postbiotics is not formally associated with them within regulation. The term postbiotics referring to inanimate microbes is emerging in some regulatory frameworks but remains to be globally incorporated. There exists an opportunity for the scientific community to serve as a resource for promoting a clear postbiotic definition and guidance on adequate analytical approaches as regulations are developed. The decision tree presented herein (Figure 1) could be a useful tool for regulators to consider to inform the development of regulations.

## 8 Conclusion

Many products that deliver inanimate microorganisms, with or without metabolites, and that conform to the ISAPP definition of postbiotics have been in the market for many years. The manufacturers of these products now have the opportunity to align their products with this new category and embark on a marketing path that utilizes this new term. It also creates opportunities for the development of new products, although challenges exist. We anticipate that a complete, full characterization and quantification of all components of a postbiotic product will not be expected. The level of characterization and quantification needed will depend on the intended recipient of the information. For measuring intact, inanimate cells, FC has evolved into a mainstream technique, with cheaper and more sophisticated instrumentation available as well as with innovations such as imaging FC. Quantitative PCR and digital PCR may be useful quantification tools as well. For cell fragments and metabolites, a panel of targeted chemical, biochemical, microbiological and immunological tests may be applied to quantify specific components associated with the activity, mechanism and/or efficacy of the postbiotic. The field of postbiotics will need to embrace multiple technological approaches to quantification, as products might vary considerably depending on their complexity, the type of microorganisms used (bacteria, yeasts, filamentous fungi), the integrity of the cell (intact vs. fragmented) and specific metabolites. No ‘gold standard’ for quantification of postbiotics in general should be expected. We hope the decision tree proposed provides useful guidance to product developers as they consider the different quantification possibilities for their products.

## Data Availability

The original contributions presented in the study are included in the article/supplementary material, further inquiries can be directed to the corresponding author.

## Footnotes

^1^ https://www.tga.gov.au/resources/resource/compositional-guidelines/akkermansia-muciniphila-strain-atcc-baa-835

^2^ https://www.nutraingredients-asia.com/Article/2025/01/20/china-association-publishes-postbiotic-count-methods/?utm_source=newsletter_daily&utm_medium=email&utm_campaign=21-Jan-2025&cid=DM1186964&bid=599110655

^3^ https://inspection.canada.ca/en/animal-health/livestock-feeds/regulatory-guidance/rg-1/chapter-3

^4^ https://www.tga.gov.au/sites/default/files/2023-01/requirements-for-microorganism-characterisation-in-listed-and-registered-complementary-medicines.pdf
